# Supplementary material for: Induced pluripotent stem cell model revealed impaired neurovascular interaction in genetic small vessel disease Cerebral Autosomal Dominant Arteriopathy with Subcortical Infarcts and Leukoencephalopathy
Source: Front Cell Neurosci. 2023 Jun 8;17:1195470. doi: 10.3389/fncel.2023.1195470 (PMC10285224; doi:10.3389/fncel.2023.1195470)
Supplement: Supplementary file 1 [file Data_Sheet_1.pdf]

Supplementary Material

Induced pluripotent stem cell model revealed impaired neurovascular interaction in genetic small vessel disease CADASIL

Wenjun Zhang, Xiangjun Zhao, Xuewei Qi, Sue J. Kimber, Nigel M. Hooper, Tao Wang*

* **Correspondence**: Corresponding Author: tao.wang@manchester.ac.uk

1. Supplementary Figures and Tables

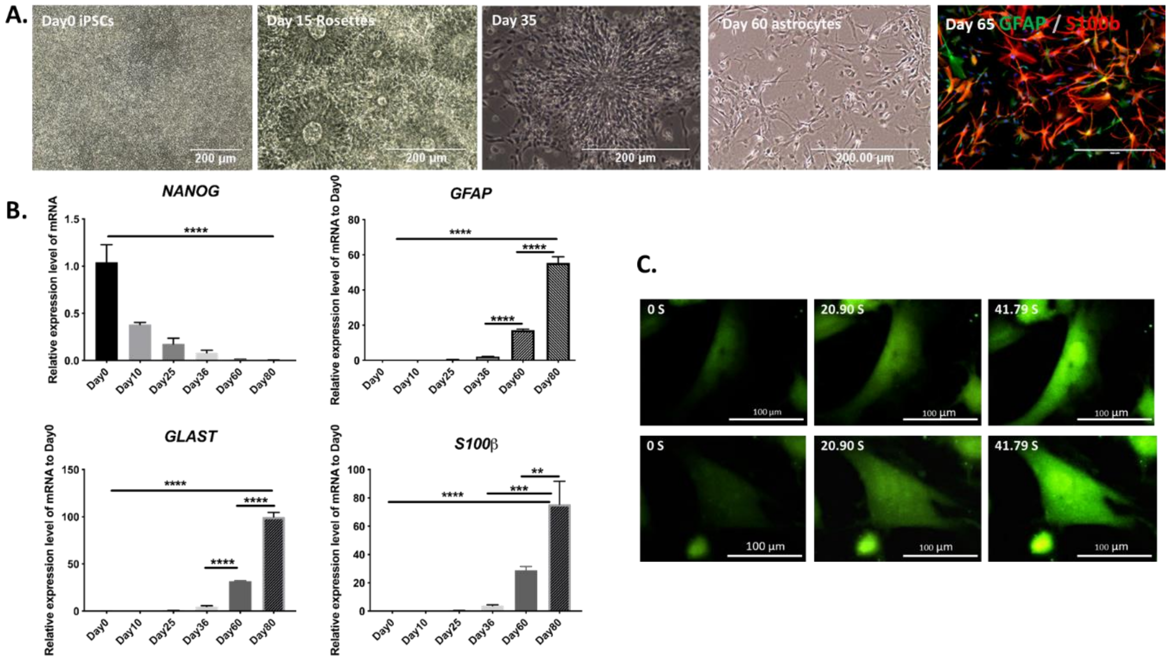

Suppl. Figure 1. **Astrocyte differentiation from iPSCs**. **A.** IPSCs were differentiated into astrocytes via a neural progenitor cell stage (neural rosettes shown on day 15 of the differentiation). Images were acquired under light microscope showing morphological changes if iPSC during differentiation up to 60 days. Immunofluorescent staining of the day 65 astrocytes showing the presence of astrocyte markers GFAP and S100b. Scale bar, 200 µm. **B.** qRT-PCR results showing the gradually reduced expression of pluripotent gene *NANOG* and increased expression of astrocyte marker genes *GFAP, GLAST* and *S100b* during the course of astrocyte differentiation from iPSCs up to 60 days. **C.** Calcium imaging showing spontaneous calcium wave in iPSC-derived astrocytes. Scale bar, 100 µm.

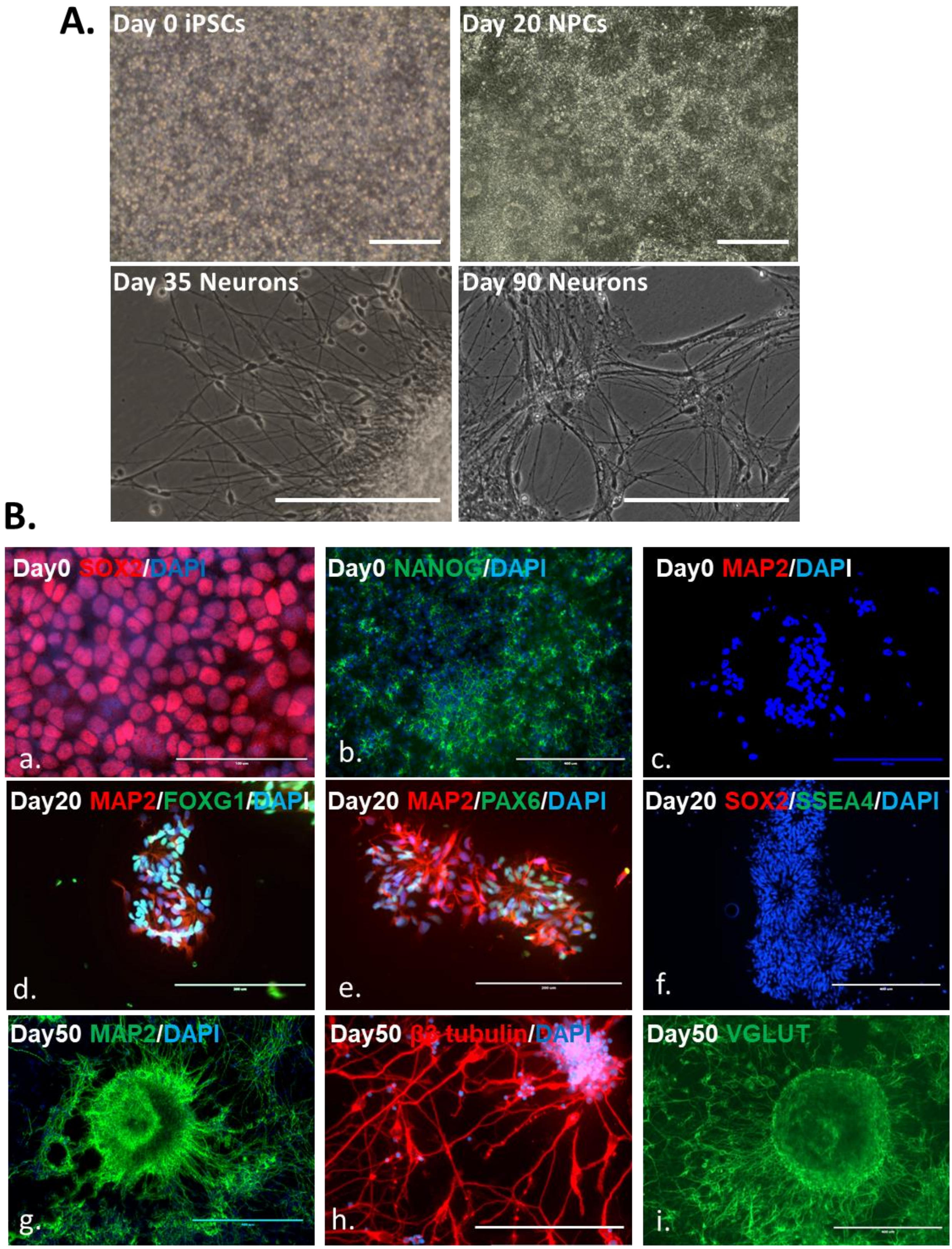

Suppl. Figure 2. **Cortical projection neuron differentiation from iPSCs**. **A.** IPSCs were differentiated into cortical projection neurons. Images taken by light microscopy shows morphological changes of the iPSC during the differentiation for up to 90 days. **B**. Immunofluorescent staining showing the presence of marker proteins in cells at different stages of the neuron differentiation. On Day 0, SOX2 and NANOG existed in iPSCs, and neural marker MAP2 was negative; on Day 20, neural progenitor cells displayed MAP, FOXG1 and PAX6, and iPSC marker SOX2 and SSEA4 were negative; on Day 50, neurons displayed neural markers of MAP2, β-tubulin and VGLUT1. Scale bars in A, B.c and B.f-i, 400 μm; in B.d, e and h, 200 μm; in B.a, 100 μm.

Suppl. Table 1. Antibodies used for immunofluorescent staining

Targets	Species	Supplier	Dilution
SOX2	Rabbit	Abcam ab181557	1:500
NANOG	Rabbit	Abcam ab109250	1:200
SSEA4	Mouse	Abcam ab16287	1:200
CD31	Mouse	Bio Techne BBA7	1:200
VE-Cadherin	Mouse	Bio Techne MAB9381	1:200
Occludin	Mouse	Life Technology 331500	1:200
Claudin 5	Mouse	Life Technology 352500	1:100
FOXG1	Mouse	Abcam ab18259	1:500
PAX6	Mouse	Covance PRB-278B	1:500
MAP2	Mouse	Abcam ab92434	1:600
β-tubulin	Rabbit	Abcam ab18207	1:500
VGLUT1	Mouse	Life Technology 33150	1:200
GFAP	Chicken	Abcam ab4674	1:600
S100β	Rabbit	Abcam ab52642	1:200
Donkey pAb anti-Rabbit Alexa Fluor® 594	Donkey	Abcam ab150064	1:500
Donkey pAb anti-Mouse Alexa Fluor® 488	Donkey	Abcam ab150105	1:500
Goat pAb anti-Chicken Alexa Fluor® 647	Donkey	Abcam ab150175	1:500
Donkey pAb anti-Goat Alexa Fluor® 488	Donkey	Abcam ab150029	1:500

Suppl. Table 2. Primer list for qRT-PCR

Target genes	Forward sequence	Reverse sequence
CLDN5	5' GTTCGCCAACATTGTCGTCC 3'	5' GTAGTTCTTCTTGTCGTAGTCGC 3'
GAPDH	5' CATGTTCGTCATGGGTGTGAACCA 3'	5' ATGGCATGGACTGTGGTCATGAGT 3'
GFAP	5' GTCCCCCACCTAGTTTGCAG 3'	5' TAGTCGTTGGCTTCGTGCTT 3'
GLAST	5' ACCCCAAGCATTCTGTGC 3'	5' TTCCGAAATAGAGCCTCGAC 3'
NANOG	5' TTAATAACCTTGGCTGCCGT 3'	5' GCAGCAAATACGAGACCTCT 3'
NOTCH3	5' CATCTCCGACCTGATCTGCC 3'	5' GTCTGTAGAGCGGTTTCGGA 3'
S100b	5' TGTAGACCCTAACCCGGAGG 3'	5' TGCATGGATGAGGAACGCAT 3'
